# State of the Art of Systemic Therapy in HPV-Positive Oropharyngeal Squamous Cell Carcinoma: A Scoping Review

**DOI:** 10.3390/diseases14020046

**Published:** 2026-01-29

**Authors:** Fausto Petrelli, Mara Ghilardi, Agostina De Stefani, Massimiliano Nardone, Vincenzo Capriotti

**Affiliations:** 1 Oncology Unit, Oncology Department, ASST Bergamo Ovest, Piazzale Ospedale 1, 24047 Treviglio, Italy; 2 Radiotherapy Unit, ASST Bergamo Ovest, 24047 Treviglio, Italy; 3 Otorhinolaryngology Unit, ASST Bergamo Ovest, 24047 Treviglio, Italy; 4 Otorhinolaryngology Unit, Mirano Hospital, 30035 Mirano, Italy

**Keywords:** HPV, HPV-positive, oropharyngeal squamous cell carcinoma, OPSCC, chemoradiation, immunotherapy, de-escalation, scoping review

## Abstract

Objectives: To synthesize current evidence and emerging data on systemic treatment strategies for early-stage and locally advanced human papillomavirus (HPV)-positive oropharyngeal squamous cell carcinoma (OPSCC), with emphasis on treatment de-escalation and the integration of immunotherapy. Data Sources: We searched PubMed/MEDLINE, Scopus, and EMBASE for English-language studies published from 2010 to 2025 using terms related to HPV-positive disease, oropharyngeal carcinoma, de-escalation, chemoradiation, and immunotherapy. Review Methods: Peer-reviewed clinical trials, meta-analyses, and key translational studies addressing systemic therapy, biomarkers, and immunotherapeutic strategies in HPV-positive OPSCC were included. Emphasis was placed on phase II–III trials evaluating cisplatin-sparing regimens, cetuximab substitution, radiation dose reduction, and early-phase immunotherapy combinations. Evidence was synthesized qualitatively. Results: Cisplatin-based concurrent chemoradiation remains the standard of care for locally advanced HPV-positive OPSCC. De-intensification trials suggest that reduced-intensity regimens may be feasible in carefully selected low-risk patients; however, replacing cisplatin with cetuximab results in inferior survival. PD-1 inhibitors (e.g., pembrolizumab, nivolumab) provide durable responses in recurrent/metastatic disease and are under active evaluation in earlier stages and in combination with therapeutic vaccines, bispecific antibodies, and viral-vector platforms. Conclusions: Systemic therapy for HPV-positive OPSCC is moving toward biomarker-informed personalization. Cisplatin-based chemoradiation remains the curative backbone, while rational de-escalation and immunotherapy integration may preserve high cure rates while reducing long-term toxicity. Ongoing phase III trials will clarify which patient subsets are most suitable for de-intensified or immunotherapeutic approaches, guiding future standards of care.

## 1. Introduction

Human papillomavirus (HPV)-positive oropharyngeal squamous cell carcinoma (OPSCC) represents one of the most significant oncologic transformations of the 21st century, evolving from a relatively rare entity to the predominant form of oropharyngeal cancer in many developed nations. Because HPV-driven head and neck cancer occurs overwhelmingly in the oropharynx, we use OPSCC as the primary term throughout this review; when included studies enroll multiple head and neck subsites, we retain the broader label of HPV-positive head and neck squamous cell carcinoma (HNSCC).

The rising incidence of HPV-associated OPSCC constitutes a global public health challenge with profound implications. Between 2000 and 2012, worldwide incidence of HPV-associated oropharyngeal squamous cell carcinoma (OPSCC) increased dramatically across North America, Europe, Japan, and Australia. This trend continues unabated, with projections indicating that by 2040, HPV-driven OPSCC incidence will increase substantially, reaching approximately 890,000 new cases annually. The United States exemplifies this shift: HPV-positive oropharyngeal cancer now accounts for approximately 70–80% of all oropharyngeal malignancies, a stark reversal from the 1970s when tobacco-related cancers predominated [1,2,3,4].

This epidemiologic transformation reflects profound changes in sexual behavior patterns, increased oral HPV transmission, and the extended latency period between initial viral exposure and malignant transformation, typically spanning 10–30 years. The median age at diagnosis for HPV-positive OPSCC patients is typically 5–10 years younger than for HPV-negative disease, with many patients presenting in their fourth or fifth decade of life. This younger age distribution has particular significance given the potential for decades of survivorship and the cumulative impact of treatment-related toxicities on quality of life [2,5].

HPV-positive OPSCC represents a biologically distinct disease entity driven primarily by high-risk HPV type 16 (HPV-16), which accounts for approximately 90–95% of cases. Compared with HPV-negative HNSCC, HPV-positive tumors generally have a lower mutational burden but show recurrent genomic alterations (e.g., PIK3CA) and characteristic immune-related gene expression patterns [5,6].

This molecular distinctiveness translates into clinically relevant phenotypic differences. HPV-positive tumors demonstrate enhanced radiosensitivity, increased immunogenicity, and distinct patterns of metastasis compared to their HPV-negative counterparts. The immune microenvironment of HPV-positive OPSCC is characterized by robust CD8+ T-cell infiltration, elevated PD-L1 expression, and a type I interferon signature, providing the biological rationale for the remarkable efficacy of immune checkpoint blockade in this disease.

Despite remarkable progress, significant challenges remain in the management of HPV-positive OPSCC. Treatment-related toxicity continues to cause substantial morbidity, particularly dysphagia, xerostomia, and neck fibrosis, which can persist for decades in these typically young survivors. The optimal management of the approximately 15–20% of patients who experience disease recurrence remains controversial, with limited evidence guiding treatment sequencing [7,8].

Furthermore, the ongoing evolution of diagnostic techniques, biomarker development, and supportive care measures necessitates a holistic approach to disease management that encompasses not only survival outcomes but also quality of life, functional preservation, and long-term survivorship issues. This review aims to provide clinicians and researchers with a comprehensive, evidence-based framework for understanding current systemic therapy options and future directions in this rapidly evolving field.

This narrative review synthesizes current evidence and emerging data on systemic treatment strategies for early and locally advanced HPV-positive OPSCC, with particular emphasis on treatment de-escalation and immunotherapy integration. Specific objectives include:

1. Evaluation of chemotherapy-based approaches and their role in curative treatment
2. Assessment of immunotherapy indications, efficacy, and limitations
3. Critical analysis of de-escalation strategies and appropriate patient selection
4. Review of emerging combination treatments and novel therapeutic approaches
5. Examination of biomarker development and personalized treatment strategies
6. Discussion of toxicity management and quality of life considerations

Through this comprehensive examination, we aim to provide clinicians with practical guidance for current practice while identifying critical knowledge gaps and future research priorities in the management of HPV-positive OPSCC.

## 2. Materials and Methods

This scoping review was conducted and reported in accordance with the PRISMA Extension for Scoping Reviews (PRISMA-ScR). The review protocol was not prospectively registered. The aim of this work was evidence mapping of contemporary systemic therapy strategies (including emerging conference evidence), rather than quantitative synthesis or guideline recommendation.

This scoping review synthesizes contemporary evidence on systemic therapy for early and locally advanced HPV-positive oropharyngeal squamous cell carcinoma (OPSCC), integrating established literature with emerging data presented at the 2025 ASCO Annual Meeting. Particular attention was devoted to chemotherapy, immunotherapy, treatment de-intensification, and combination strategies, with the overarching goal of contextualizing evolving therapeutic paradigms within the biological distinctiveness of HPV-positive disease.

### 2.1. Literature Search and Inclusion Criteria

A comprehensive literature search was undertaken following the SANRA (Scale for the Assessment of Narrative Review Articles) methodological framework, ensuring transparency, reproducibility, and rigor appropriate for a narrative synthesis. Searches were conducted across PubMed, Scopus, and EMBASE from January 2015 through June 2025 to capture a decade of rapidly expanding evidence in HPV-related HNSCC. Additional manual searches included screening of reference lists from major review articles, meta-analyses, and consensus guidelines, as well as abstracts and oral presentations from the 2025 ASCO Annual Meeting.

Multiple combinations of predefined keywords and Boolean operators were used to identify eligible studies. Search terms included “HPV-positive”, “oropharyngeal cancer” OR “OPSCC”, “head and neck squamous cell carcinoma”, “chemoradiation”, “de-escalation”, “immunotherapy”, and “treatment intensification”, with variations and Medical Subject Headings (MeSH) where applicable. The final search was performed on 30 June 2025.

Studies were eligible for inclusion if they met the following criteria:

- Reported systemic therapy outcomes (chemotherapy, immunotherapy, or combination treatments) specifically in HPV-positive OPSCC.
- Were published between 2015 and 2025 in peer-reviewed journals.
- Included randomized or non-randomized clinical trials, meta-analyses, observational studies, or translational biomarker analyses relevant to systemic treatment.
- Presented data pertaining to early-stage, locally advanced, or recurrent/metastatic HPV-positive OPSCC.
- Included abstracts or oral presentations from major oncology meetings (e.g., ASCO 2025) reporting relevant clinical or translational findings.

Exclusion criteria were:

- Studies exclusively evaluating HPV-negative HNSCC or non-squamous histologies.
- Preclinical, animal, in vitro, or purely mechanistic laboratory studies lacking clinical correlation.
- Articles unrelated to systemic therapy (e.g., surgical techniques, radiotherapy-only studies, diagnostic performance analyses).
- Non-English publications, case reports, or expert opinions without data.

This search strategy ensured the inclusion of high-impact trials, pivotal translational studies, and recent innovative research informing current trends in systemic management of HPV-positive disease. The search was intentionally restricted to January 2015–June 2025 to map evidence that is most applicable to contemporary management of HPV-positive OPSCC. This interval captures (i) the modern IMRT era and broadly contemporary supportive-care standards, improving comparability of functional and toxicity outcomes; (ii) more consistent HPV attribution and risk stratification (routine p16-based classification and contemporary staging/selection approaches), enabling clearer interpretation of de-intensification eligibility; and (iii) the key therapeutic developments motivating this scoping review—publication of pivotal randomized trials evaluating cisplatin-sparing or de-escalation strategies and the maturation of immune checkpoint blockade in recurrent/metastatic disease with rapid expansion into curative-intent combinations.

### 2.2. Data Extraction and Synthesis

Data extraction was performed independently by two reviewers (FP and VC), with discrepancies resolved by consensus. For each study, the following elements were recorded: study design, sample size, HPV testing method (p16, DNA/RNA-based assays), disease setting (early, locally advanced, recurrent/metastatic), systemic therapy regimen, treatment intensity (standard vs. de-escalated), radiation details (when applicable), primary and secondary endpoints, survival outcomes (OS, PFS), response rates, toxicity profiles, and biomarker findings.

Studies were then categorized into major thematic domains reflecting key therapeutic strategies:

- Chemotherapy and Chemoradiation: Evidence supporting cisplatin-based chemoradiation, alternatives for cisplatin-ineligible patients, and emerging data on systemic de-escalation, including induction chemotherapy and radiation dose reduction.
- Immunotherapy: Clinical trials of PD-1 inhibitors (pembrolizumab, nivolumab) in recurrent/metastatic HPV-positive OPSCC; early-phase trials of vaccine-based and viral-vector therapies; and combinations of immunotherapy with radiation or chemotherapy.
- De-escalation Strategies: Reduced radiation doses, omission of chemotherapy, adaptive treatment approaches based on tumor biology or response, and stratification by smoking status or tumor burden.
- Combination Therapies: Novel combinations of checkpoint inhibitors, therapeutic HPV vaccines, bispecific antibodies, targeted therapies, and chemoradiation platforms.

Particular emphasis was placed on studies from ASCO 2025, including early efficacy and safety results of VERSATILE-002, HB-200 combination programs, and ongoing de-escalation trials (e.g., NRG-HN005, OPTIMA, and institutional TORS-guided approaches).

Synthesis followed a qualitative thematic approach. Given the heterogeneity of study designs and endpoints, no quantitative meta-analysis was performed. Instead, narrative integration allowed contextual comparison of treatment strategies across risk groups, disease stages, and therapeutic modalities.

### 2.3. Assessment of Clinical Relevance

The clinical relevance of each systemic strategy was evaluated by assessing its applicability to both early and advanced HPV-positive disease. Outcome measures included oncologic endpoints (OS, PFS, locoregional control), functional parameters (swallowing, speech, toxicity burden), and feasibility of integrating systemic therapy with radiation or surgery.

Particular attention was given to:

- Oncologic efficacy in comparison with standard cisplatin-based chemoradiation.
- Toxicity reduction as a major driver of de-escalation in a young population with long life expectancy.
- Biomarker-guided personalization, including circulating HPV DNA, PD-L1 expression, and immune infiltration signatures.
- Real-world generalizability, using retrospective and observational cohorts that reflect broader clinical practice beyond trial-selected populations.
- Translational implications of emerging immunotherapy-based strategies, vaccine platforms, and bispecific antibodies.

The GRADE framework was applied to categorize the certainty of evidence for key treatment approaches, including high-quality randomized trials (strong evidence), moderate-quality cohort or translational studies, and low-certainty early-phase data. This structured evaluation supports transparent interpretation of the strength of recommendations and highlights areas where further randomized evidence is required.

In the **GRADE** framework, the certainty (quality) of evidence is commonly displayed with **“plus” symbols** (often shown as filled circles “⊕” in publications). The mapping is:

- **⊕⊕⊕⊕ High certainty**
  We are very confident the true effect is close to the estimate. Further research is very unlikely to change the estimate.
- **⊕⊕⊕◯ Moderate certainty**
  The true effect is probably close to the estimate, but further research may change the estimate.
- **⊕⊕◯◯ Low certainty**
  The true effect may be substantially different from the estimate; further research is likely to change the estimate.
- **⊕◯◯◯ Very low certainty**
  The true effect is likely substantially different from the estimate; the estimate is very uncertain.

### 2.4. Risk of Bias and Certainty of Evidence

Risk of bias (RoB) was assessed at the study level for included comparative clinical evidence. Randomized trials were appraised using the Cochrane RoB 2 tool, and non-randomized comparative studies were appraised using ROBINS-I. For conference abstracts lacking sufficient methodological detail for formal appraisal, we prespecified a conservative approach, treating these sources as having a high risk of reporting bias and using them primarily for horizon scanning rather than practice-changing inference.

Certainty of evidence was then summarized at the strategy level using a GRADE-informed approach. We prioritized clinically relevant outcomes. Randomized evidence started at high certainty and could be rated down for RoB, inconsistency, indirectness, imprecision, or suspected publication/reporting bias. Observational evidence started at low certainty and could be rated up only in the presence of compelling signals (e.g., large effects) with acceptable risk of bias. Final certainty ratings for each strategy are presented in Table 1 and Table 2 together with RoB judgments and are intended to support transparent evidence mapping and identification of knowledge gaps.

## 3. Results

### 3.1. Study Overview

**Figure 1:**
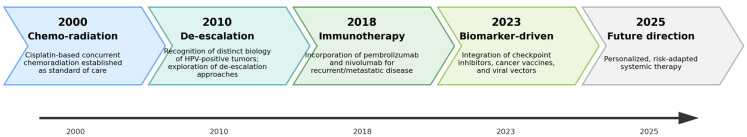
Evolution of systemic therapy strategies in HPV-positive oropharyngeal squamous cell carcinoma (OPSCC).

**Figure 2:**
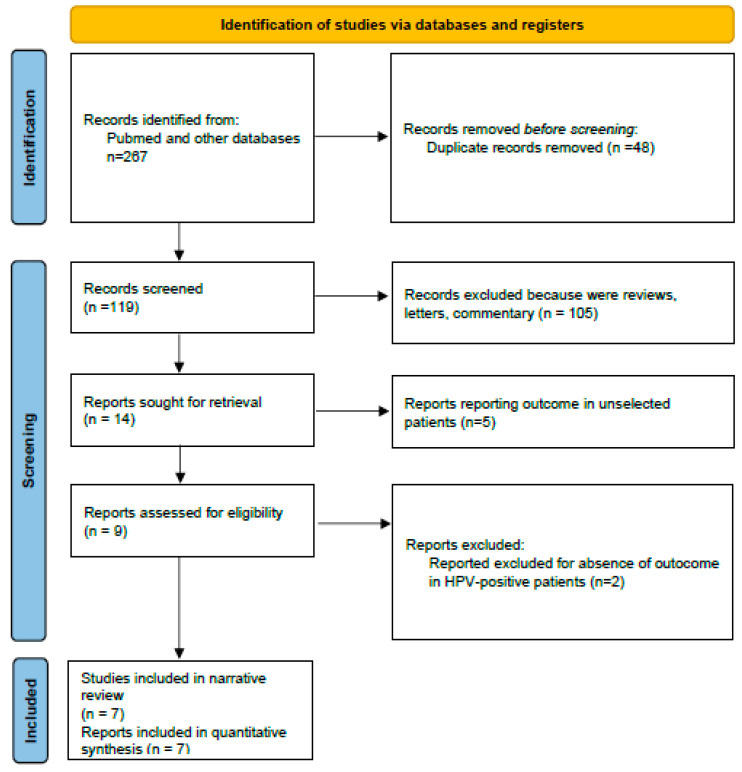
Flow diagram of included studies.

In total, 267 articles were initially retrieved. After removal of duplicates and relevance screening, 114 full-text articles were assessed for eligibility. Of these, 67 studies were included in the review, comprising: 23 randomized clinical trials (phase II–III), 19 review or meta-analysis papers, 15 translational or biomarker studies, and 10 meeting abstracts (including ASCO 2025 oral presentations). Finally, 7 pivotal studies were selected for comment in this review (Figure 1 and Figure 2; Table 3) [9,10,11,12,13,14,15].

### 3.2. Epidemiology and Pathogenesis

HPV-related oropharyngeal cancers, predominantly driven by HPV type 16, represent a growing proportion of head and neck cancers. The pathogenesis involves viral genome integration into host cell DNA, resulting in overexpression of viral oncoproteins E6 and E7. These proteins inactivate tumor suppressors p53 and retinoblastoma (Rb), disrupting cell cycle control and promoting malignant transformation. HPV-positive tumors are typically more radiosensitive and elicit stronger immune responses, contributing to their favorable prognosis relative to HPV-negative counterparts.

### 3.3. Early-Stage HPV-Positive OPSCC

Treatment paradigms are shifting towards de-intensification strategies aimed at reducing treatment-related toxicities without compromising oncologic outcomes. HPV-positive tumors’ increased sensitivity to radiation and chemotherapy allows for reduced doses and treatment volumes. However, no treatment de-escalation can be recommended outside of clinical trials.

**Chemotherapy:** Systemic chemotherapy is rarely indicated in early-stage disease. Surgery or radiation alone is generally sufficient. Induction chemotherapy (cisplatin, docetaxel, or 5-FU) may reduce tumor burden, but its benefit remains unproven. Adjuvant chemotherapy has limited application, with trials suggesting minimal survival benefit in low-risk patients.

**De-escalation Trials:** The De-ESCALaTE HPV trial showed that radiation dose reduction and substitution of cisplatin with cetuximab resulted in inferior survival. The NRG-HN005 trial supported selective dose reduction in favorable-risk groups without compromising outcomes. Ongoing efforts to stratify treatment based on biomarkers such as p16INK4a and circulating HPV DNA are crucial to identify candidates for safe de-escalation while minimizing long-term toxicities.

### 3.4. Locally Advanced HPV-Positive OPSCC

In advanced-stage or recurrent/metastatic disease, systemic therapy aims to prolong survival, control disease progression, and alleviate symptoms. Platinum-based chemotherapy (cisplatin or carboplatin) combined with 5-fluorouracil and/or a taxane remains standard first-line treatment. Cetuximab incorporation has shown improved survival outcomes in certain populations, though its benefit in HPV-positive tumors may be less pronounced.

**Chemoradiation:** Cisplatin remains the agent of choice due to its radiosensitizing effect and survival benefit. In cisplatin-ineligible patients, alternatives such as carboplatin or docetaxel may be used, though often less effective.

**Immunotherapy:** Checkpoint inhibitors have gained traction in both recurrent/metastatic and locally advanced settings. Pembrolizumab demonstrated robust responses in recurrent/metastatic HNSCC, particularly in PD-L1–positive tumors (KEYNOTE-048 trial). ASCO 2025 data suggest immunotherapy may benefit earlier-stage disease: the VERSATILE-002 trial (pembrolizumab + PDS0101 vaccine) reported durable responses in HPV16-positive tumors. Novel platforms like HB-200 (viral vector immunotherapy) and ficerafusp alfa (EGFR/TGF-β bispecific antibody) are being explored in combination with ICIs.

**Personalized Medicine:** Treatment personalization is gaining momentum. Beyond p16, emerging biomarkers include circulating HPV DNA (for risk stratification and therapy monitoring), PD-L1 expression and tumor mutational burden (TMB) as predictors of ICI response, and immune infiltration signatures. A precision medicine approach integrating these molecular indicators with clinical staging could enhance outcomes and reduce toxicity.

### 3.5. Evidence Synthesis (Table 2 and Table 3)

#### 3.5.1. Cisplatin-Based Concurrent Chemoradiation (GRADE ⊕⊕⊕⊕ High Certainty)

The most robust evidence supports **cisplatin-based concurrent chemoradiation (CRT)** as the reference standard for **locally advanced HPV-positive (p16+) oropharyngeal squamous cell carcinoma (OPSCC)** in patients eligible for cisplatin. The evidence base is anchored by two concordant phase III trials demonstrating that cisplatin should not be replaced by cetuximab in this setting. In **RTOG 1016** (n ≈ 850), cetuximab-RT failed non-inferiority and was associated with worse overall survival (OS) (HR for death 1.45; 5-year OS ~84.6% with cisplatin vs. ~77.9% with cetuximab) and worse progression-free survival (PFS) (HR 1.72). In **De-ESCALaTE HPV** (n = 334), 2-year OS was significantly higher with cisplatin versus cetuximab (97.5% vs. 89.4%; HR 4.99). Meta-analytic syntheses in HPV-positive OPSCC similarly confirm inferior OS/PFS with cetuximab substitution compared with cisplatin [9,10].

**Clinical implication:** Cisplatin-based CRT remains the definitive standard for fit patients with locally advanced HPV-positive disease.

#### 3.5.2. Cetuximab Substitution for Cisplatin (GRADE ⊕⊕⊕⊕ High Certainty for Inferiority)

High-level evidence indicates that **cetuximab substitution** for cisplatin during definitive RT results in **inferior oncologic outcomes**, without a compensatory improvement in meaningful long-term quality of life. Across both RTOG 1016 and De-ESCALaTE HPV, cetuximab-RT was associated with significantly worse OS and disease control compared with cisplatin-RT.

**Clinical implication:** Cetuximab should be reserved for **cisplatin-ineligible** patients (e.g., severe baseline hearing loss, renal impairment, or other contraindications), recognizing the trade-off in efficacy.

#### 3.5.3. Pembrolizumab Immunotherapy in Recurrent/Metastatic Disease (GRADE ⊕⊕⊕⊕ High Certainty)

Immune checkpoint blockade with pembrolizumab has high-quality evidence supporting use in **first-line recurrent/metastatic (R/M) HNSCC**. In **KEYNOTE-048**, pembrolizumab monotherapy improved OS versus cetuximab-chemotherapy in PD-L1 CPS–enriched populations (e.g., CPS ≥ 20: HR 0.61), while pembrolizumab chemotherapy improved OS across CPS-defined and total populations (e.g., CPS ≥ 20: HR 0.62; total population: HR 0.71). Long-term analyses show a survival “tail” with pembrolizumab monotherapy becoming apparent around the 4-year landmark. More mature follow-up (≈5–6 years) continues to support durable benefit of pembrolizumab-based strategies versus EXTREME. In addition, post hoc analyses indicate preserved feasibility of subsequent-line therapies after pembrolizumab exposure. [11,16,17,18]

**Clinical implication:** Pembrolizumab (alone for appropriately selected PD-L1–positive patients, or combined with chemotherapy when rapid response is needed) is a first-line standard in R/M HNSCC.

#### 3.5.4. De-Escalation Strategies in HPV-Positive OPSCC (GRADE ⊕⊕⊕◯ Moderate Certainty)

Evidence supporting **treatment de-intensification** remains moderate and is best viewed as **strategy-dependent**:

(1) **Dose-reduction de-escalation (definitive CRT):** Randomized evidence has cautioned against unselected dose de-escalation. In **NRG-HN005** (p16+ OPSCC, ≤10 pack-years), the reduced-dose arms did not meet non-inferiority; 2-year PFS was 98.1% in the control arm versus 88.6% (60 Gy + cisplatin) and 90.3% (60 Gy + nivolumab) [13].
(2) **Response-adapted approaches after induction chemotherapy: QUARTERBACK** tested response-adapted de-intensification using induction TPF followed by standard-dose CRT (70 Gy) versus reduced-dose CRT (56 Gy) with weekly carboplatin among responders. Long-term QoL analyses favored reduced-dose CRT in several domains, while long-term disease-control endpoints appeared comparable in this selected population [15].
(3) **Surgery/TORS with risk-adapted adjuvant therapy:** PATHOS is a pivotal platform evaluating post-operative risk stratification to reduce adjuvant RT/CRT intensity while preserving outcomes. Importantly, PATHOS recruitment has closed (October 2024) with >1300 participants registered, supporting feasibility and anticipated maturity of comparative outcomes [19].

The molecular pathogenesis of HPV-positive OPSCC differs fundamentally from HPV-negative disease. High-risk HPV infection (predominantly HPV-16) is associated with a comparatively low mutational burden, recurrent genomic alterations, and a distinct tumor immune microenvironment. Viral antigen expression, CD8+ T-cell infiltration, interferon signaling, and PD-L1 upregulation may contribute to improved treatment response and favorable prognosis, and provide a biologic rationale for immune checkpoint blockade in recurrent/metastatic OPSCC [19,20,21,22].

## 4. Discussion

HPV-positive head and neck squamous cell carcinoma (HNSCC), particularly oropharyngeal squamous cell carcinoma (OPSCC), has emerged as a biologically and clinically distinct disease entity. Its rising incidence among younger, non-smoking individuals contrasts with the decline of HPV-negative tumors, redefining the landscape of head and neck oncology. This epidemiologic transformation likely reflects changing sexual behaviors, increased oral HPV transmission, and the long latency between viral exposure and malignant transformation [23,24,25]. The median age at diagnosis for HPV-positive OPSCC is typically younger than for HPV-negative disease, with many patients presenting in their fifth or sixth decade of life—when functional preservation is particularly critical [23,24].

The molecular pathogenesis of HPV-positive OPSCC differs fundamentally from its HPV-negative counterpart. High-risk HPV types, predominantly HPV-16, integrate into the host genome and express viral oncoproteins E6 and E7, which inactivate tumor suppressors p53 and retinoblastoma (Rb), respectively [23,26,27,28,29]. This viral-mediated carcinogenesis yields tumors with distinct genomic profiles and fewer chromosomal alterations than tobacco/alcohol-related HNSCC.These molecular features may contribute to enhanced radiosensitivity and improved treatment response [23,30]. Furthermore, viral antigen expression creates an immunogenic tumor microenvironment enriched with tumor-infiltrating lymphocytes (particularly CD8+ T cells), which is associated with favorable prognosis and provides a biologic rationale for immunotherapy sensitivity [19,21,22,31,32,33].

This epidemiologic shift carries major implications for otorhinolaryngologists, who are often the first specialists to evaluate persistent cervical lymphadenopathy, tonsillar asymmetry, or oropharyngeal ulceration. Early clinical suspicion and HPV testing have become integral to diagnostic pathways, facilitating accurate prognostication and tailored treatment planning within multidisciplinary tumor boards. The typical presentation of HPV-positive OPSCC often includes cystic cervical lymph nodes—sometimes the only clinically apparent manifestation—requiring thorough examination of Waldeyer’s ring and consideration of fine-needle aspiration with HPV testing when the primary site is not immediately evident. Otorhinolaryngologists must maintain high clinical vigilance, as early detection significantly impacts treatment options and outcomes. The integration of narrow-band imaging and other advanced endoscopic techniques has enhanced the ability to identify subtle mucosal changes in the oropharynx, improving diagnostic accuracy and enabling earlier intervention.

Systemic therapy in HPV-positive OPSCC has evolved from uniform chemoradiation toward risk-adapted, biomarker-informed strategies. Cisplatin-based concurrent chemoradiation remains the cornerstone for locally advanced disease; however, its recognized long-term toxicity profile has driven de-intensification trials aiming to maintain oncologic control while reducing dysphagia, xerostomia, and ototoxicity—side effects that critically affect speech and swallowing, domains central to ENT care. De-escalation approaches, such as those tested in RTOG 1016 and De-ESCALaTE HPV, confirmed that cetuximab cannot replace cisplatin in fit patients, but selective dose reduction in low-risk cases may be feasible. These landmark trials established that while treatment de-intensification is conceptually appealing, it must be pursued cautiously with rigorous patient selection criteria. Current de-escalation strategies under investigation include reduced radiation doses (from 70 Gy to 60 Gy), omission of concurrent chemotherapy in selected low-risk patients, and substitution of cisplatin with less toxic alternatives in specific clinical contexts. The challenge lies in identifying reliable predictive biomarkers that can prospectively identify patients who will maintain excellent outcomes with reduced treatment intensity [23,30,34,35].

Risk stratification has become increasingly sophisticated, incorporating not only HPV status but also smoking history, nodal burden, T-stage, and emerging molecular markers. Patients with minimal or no smoking history, limited nodal disease (N0-N2a), and smaller primary tumors (T1–T2) represent a favorable-risk cohort that may be candidates for de-escalation protocols. Conversely, patients with heavy smoking history (>10 pack-years), bulky nodal disease, or advanced T-stage require standard-intensity treatment despite HPV-positive status, as their outcomes approach those of HPV-negative disease. This nuanced risk stratification underscores the heterogeneity within HPV-positive OPSCC and the need for individualized treatment planning that extends beyond simple HPV status determination.

A multidisciplinary approach—uniting otorhinolaryngologists, oncologists, radiotherapists, pathologists, and speech therapists—is indispensable. Surgeons play a key role in diagnostic biopsies, transoral robotic or laser resections, and management of treatment sequelae. Collaboration with medical oncologists ensures optimal timing of systemic therapy, while rehabilitation specialists support post-treatment recovery. Close multidisciplinary integration has been shown to improve both survival and functional outcomes in HPV-positive OPSCC. Regular tumor board meetings facilitate comprehensive case review, enabling treatment decisions that balance oncologic efficacy with functional preservation and quality of life considerations. The complexity of modern head and neck cancer care demands seamless communication across specialties, with each discipline contributing unique expertise to optimize patient outcomes. Nutritional support, dental evaluation, and psychosocial counseling should be integrated early in the treatment pathway, as these supportive measures significantly impact treatment tolerance and long-term recovery.

Immunotherapy has redefined the management of recurrent or metastatic disease. Agents targeting the PD-1/PD-L1 axis, including pembrolizumab and nivolumab, have demonstrated clinically meaningful and in some cases durable benefit compared with historical cytotoxic approaches, alongside patient-reported outcome advantages in contemporary studies [18,34,36,37,38,39,40]. Recent studies exploring the integration of immunotherapy into earlier stages and/or in combination with vaccines or bispecific antibodies are ongoing. [41,42] For ENT specialists, these advances underline the growing need to coordinate systemic therapy with local and functional management (airway protection, swallowing rehabilitation) and toxicity surveillance [21,42].

The KEYNOTE-048 trial established pembrolizumab-based regimens as first-line therapy for recurrent/metastatic HNSCC, with greatest benefit in PD-L1–positive subgroups. Durable disease control with immunotherapy represents a paradigm shift from conventional chemotherapy, but immune-related adverse events require vigilant monitoring and prompt management, including education on toxicities ranging from dermatologic events to pneumonitis, colitis, and endocrinopathies [21,42,43].

Surgical innovations, particularly transoral robotic surgery (TORS), have expanded the therapeutic armamentarium in selected early-stage HPV-positive OPSCC. In appropriately staged patients, TORS can achieve excellent oncologic outcomes with favorable functional results and may facilitate adjuvant de-intensification in pathologically low-risk cases [36,44,45,46,47]. Patient selection remains critical, and comparative data (e.g., ORATOR) suggest similar survival with differing functional recovery trajectories between surgery-based and definitive (chemo)radiation approaches [36].

Emerging biomarkers—such as circulating HPV DNA, PD-L1 expression, and composite immune signatures—may refine patient selection for de-escalated or immunotherapy-based protocols [19,37,38,39,40,48,49,50]. Circulating HPV DNA is particularly promising for response monitoring and early recurrence detection, enabling non-invasive longitudinal surveillance and potentially adaptive treatment strategies [37]. As survival improves, structured survivorship programs should address long-term toxicities (dysphagia, xerostomia, fibrosis) and psychosocial sequelae within multidisciplinary pathways [23,36,44,45,46,47].

Global implementation of HPV vaccination remains a cornerstone of primary prevention, and ENT clinicians are key advocates for vaccination awareness and early detection. The introduction of prophylactic HPV vaccines (bivalent, quadrivalent, and nonavalent formulations) has demonstrated remarkable efficacy in preventing HPV infection and associated precancerous lesions. Population-level data from countries with high vaccination coverage, such as Australia and Scotland, have shown significant reductions in HPV prevalence and early evidence of declining oropharyngeal cancer incidence in vaccinated cohorts. However, global vaccination coverage remains suboptimal, with significant disparities between high- and low-resource settings. Achieving the World Health Organization’s goal of 90% vaccination coverage by 2030 requires concerted efforts to address vaccine hesitancy, improve access, and implement school-based vaccination programs. Equitable access to vaccination, molecular testing, and clinical trials must be prioritized to reduce outcome disparities between high- and low-resource settings. ENT specialists, as frontline clinicians encountering the consequences of HPV-related disease, have a unique platform to advocate for vaccination and educate patients, families, and communities about prevention strategies.

Future research should prioritize: (1) prospective validation of circulating HPV DNA as a dynamic biomarker for minimal residual disease and surveillance, with standardized assays and clinically validated thresholds for treatment decisions; (2) integration of immunotherapy into curative settings using composite oncologic and functional endpoints that capture both survival and quality of life outcomes; (3) adaptive trial designs enabling rapid evaluation of novel combinations such as therapeutic vaccines targeting HPV oncoproteins, oncolytic viral vectors, and bispecific antibodies that redirect T cells to tumor cells; (4) harmonization of PD-L1 and HPV testing methodologies to ensure reproducibility across institutions and enable meaningful comparison of trial results; and (5) policies promoting equitable access to vaccination, diagnostics, and immunotherapies worldwide, with particular attention to low- and middle-income countries where the burden of HPV-related disease is projected to increase most dramatically. Additionally, research into the tumor immune microenvironment, mechanisms of treatment resistance, and optimal sequencing of therapies will be essential to further improve outcomes.

## 5. Conclusions

This review has several limitations that should be acknowledged. Although a structured literature search across multiple databases was performed to enhance transparency and reproducibility, this work was designed as a scoping review rather than a formal systematic review. Consequently, the review protocol was not prospectively registered, and no formal risk-of-bias assessment or quantitative meta-analysis was undertaken. The included evidence is heterogeneous in terms of study design, patient populations, disease stages, therapeutic strategies, and clinical endpoints, encompassing randomized and non-randomized trials, early-phase studies, translational research, and recent conference presentations. This heterogeneity precludes pooled effect estimation and limits direct comparative conclusions. In addition, study selection and qualitative synthesis, while performed systematically, may be subject to inherent selection bias typical of narrative and scoping approaches. Finally, emerging data—particularly from ongoing phase II–III trials and recent scientific meetings—are immature and should be interpreted cautiously. Accordingly, the findings of this review should be viewed as a comprehensive mapping of current and evolving systemic therapy strategies in HPV-positive OPSCC rather than as definitive evidence to support specific treatment recommendations outside established standards of care.

In summary, systemic therapy for HPV-positive OPSCC is entering an era of personalized, multidisciplinary care. Otorhinolaryngologists occupy a central position in this evolving paradigm—bridging diagnosis, functional preservation, and collaboration with oncology and radiotherapy teams. Future research should validate circulating HPV DNA as a real-time biomarker, standardize functional endpoints (swallowing, speech), and further integrate immunotherapy into curative-intent strategies. Through coordinated multidisciplinary management, the field can achieve both oncologic excellence and preservation of quality of life in this growing patient population. The next decade promises continued refinement of risk-adapted treatment strategies, broader implementation of immunotherapy in earlier disease stages, and hopefully, the beginning of measurable impact from population-level HPV vaccination programs on disease incidence. Success will require sustained collaboration across disciplines, continued investment in translational research, and unwavering commitment to patient-centered care that prioritizes both survival and quality of life.

## Figures and Tables

**Table 1:** Systemic Therapy Modalities in HPV-positive Head and Neck Squamous Cell Carcinoma.

Therapy Modality	Key Agents/ Regimens	Clinical Setting	Evidence Level	Major Clinical Trials	Key Outcomes	Advantages	Limitations/Toxicities
Chemotherapy	• Weekly or 3-weekly cisplatin •Carboplatin (AUC 5) • 5-Fluorouracil (1000 mg/m^2^/d × 4d) • Docetaxel, Paclitaxel	• Concurrent with RT (LA disease) • Induction chemotherapy • Recurrent/metastatic setting	GRADE ●●●● (high)	• RTOG 1016 •De-ESCALaTE • TAX 324/323 • EORTC 24971	• Cisplatin concurrent: 5-year OS ~85% • Superior to cetuximab substitution • Standard of care for LA disease	• Established efficacy • Curative potential • Cost-effective	• Nephrotoxicity, ototoxicity • Nausea/vomiting • Myelosuppression • Not suitable for all patients
De-escalation Strategies	• Reduced RT dose (50–60 Gy) • Substituted agents • Shorter treatment duration • Biomarker-guided approaches	• Low-risk HPV+ patients • Clinical trials only • T1-2, N0-1, non-smokers	GRADE ●●●○ (moderate)	• PATHOS (Phase III) • NRG HN-005 (Phase II/III) • Quarterback 2b • MC1273	• Ongoing trials • Aims to reduce toxicity while maintaining efficacy • Careful patient selection crucial	• Reduced acute/late toxicity • Improved quality of life • Potential cost savings	• Not standard of care • Risk of reduced efficacy • Requires careful patient selection • Limited long-term data
Targeted Therapy	• Cetuximab (400 mg/m^2^ loading, 250 mg/m^2^ weekly) • EGFR inhibitors	• Cisplatin-ineligible patients • R/M disease • Combination approaches	GRADE ●●●○ (moderate)	• EXTREME trial • RTOG 1016 (vs. cisplatin)	• Inferior to cisplatin in HPV+ disease • OS detriment in RTOG 1016 • Option for cisplatin-ineligible	•Non-nephrotoxic • Different toxicity profile • Oral options available	• Skin toxicity • Infusion reactions • Less effective in HPV+ disease • Higher cost than chemotherapy

LA = locally advanced, R/M = recurrent/metastatic, OS = overall survival, RT = radiation therapy, HPV = human papillomavirus, AUC = area under the curve.

**Table 2:** Certainty of evidence (GRADE) and risk of bias (RoB) for key systemic-therapy strategies in HPV-positive OPSCC/HNSCC.

Therapeutic Strategy (PICO Shorthand)	Setting/Population	Key Evidence Base (Representative)	RoB Tool (By Design)	Overall RoB Judgement	Main RoB Drivers/Notes	Certainty of Evidence (GRADE)
Cisplatin-based concurrent CRT (cisplatin-RT vs. alternative systemic intensities)	Locally advanced HPV+ OPSCC; cisplatin-eligible	RTOG 1016; De-ESCALaTE HPV (comparative backbone trials establishing standard).	RoB 2 (RCTs)	Low (for OS/PFS)	Randomization robust; endpoints largely objective (OS/PFS). Open-label design may introduce some concerns for subjective outcomes (e.g., QoL), but not typically for mortality.	⊕⊕⊕⊕ High
Cetuximab substitution for cisplatin (cetuximab-RT vs. cisplatin-RT)	Locally advanced HPV+ OPSCC	RTOG 1016; De-ESCALaTE HPV show inferiority of substitution.	RoB 2 (RCTs)	Low (inferiority for OS)	Same considerations as above; inferiority signal consistent across RCTs; objective endpoints.	⊕⊕⊕⊕ High (inferiority)
Pembrolizumab ± chemotherapy (1L) (pembro±chemo vs. cetuximab+chemo)	Recurrent/metastatic HNSCC (includes HPV-associated OPSCC)	KEYNOTE-048 (phase III).	RoB 2 (RCT)	Low (for OS)	Large RCT; OS objective. Open-label may affect treatment switching and patient-reported outcomes; interpret QoL endpoints with caution if emphasized.	⊕⊕⊕⊕ High
Nivolumab (post-platinum) (nivolumab vs. investigator’s choice)	R/M HNSCC after platinum	CheckMate 141 (phase III).	RoB 2 (RCT)	Low (for OS)	RCT with OS endpoint; similar open-label caveat for subjective outcomes.	⊕⊕⊕⊕ High
De-escalation strategies (dose-reduced RT/CRT; response-adapted after induction; post-TORS risk-adapted adjuvant)	“Low-risk” HPV+ OPSCC; trial-only	NRG-HN005 interim futility; QUARTERBACK; PATHOS (platform; outcomes pending).	RoB 2 (RCTs)/ROBINS-I (non-randomized)	Some concerns → High (overall, across strategies)	Heterogeneous designs/endpoints; interim analyses and ongoing trials; response-adapted approaches increase risk of selection/performance bias; non-randomized cohorts add confounding.	⊕⊕⊕◯ Moderate
Targeted therapy in cisplatin-ineligible or R/M (EGFR-based regimens as alternative)	Cisplatin-ineligible LA disease and/or R/M	EXTREME (context), RTOG 1016 informs limits of cetuximab substitution in HPV+ curative setting.	RoB 2/indirectness note	Some concerns	Evidence may be indirect for HPV+ OPSCC specifically (mixed HNSCC populations; different comparators); treatment selection bias in cisplatin-ineligible cohorts.	⊕⊕⊕◯ Moderate
Novel ICI combinations (vaccines/viral vectors/bispecifics)	Mostly R/M HPV16+ programs; early integration in earlier stages	ASCO 2025 early-phase programs (e.g., VERSATILE-002, HB-200 combinations; phase 1/2 cohorts).	ROBINS-I (non-randomized)/“conference evidence” flag	Serious/Critical (typical)	Early-phase, often single-arm; small samples; immature follow-up; selective reporting risk higher for conference abstracts.	⊕⊕◯◯ Low

Graph legend: ⊕⊕⊕⊕ high; ⊕⊕⊕◯ moderate; ⊕⊕◯◯ low. The manuscript already defines the GRADE mapping and states it was applied to categorize certainty.

**Table 3:** Description of early-phase and pivotal studies in HPV-positive HNSCC.

Trial Name	Phase	Population	Intervention	Comparison	Key Results	Status/Implications
KEYNOTE-048	Phase III	R/M HNSCC (n = 882)	Pembrolizumab ± chemo	Cetuximab + chemo	OS benefit: HR 0.61 (CPS ≥ 20)	First-line standard for PD-L1+
RTOG 1016	Phase III	LA HPV+ OPSCC (n = 849)	RT + cetuximab	RT + cisplatin	Inferior OS: HR 1.72	Confirms cisplatin superiority
De-ESCALaTE	Phase III	LA HPV+ OPSCC (n = 334)	RT + cetuximab	RT + cisplatin	Inferior survival (*p* = 0.0007)	Cetuximab not recommended
PATHOS	Phase III	Post-TORS HPV+ (n = 1100)	De-escalated adjuvant	Standard adjuvant	Ongoing	Largest de-escalation trial
NRG HN-005	Phase II/III	Low-risk HPV+ (n = 500)	Reduced dose RT + nivolumab	Standard RT + cisplatin	Ongoing	Immunotherapy de-escalation
CheckMate 141	Phase III	R/M HNSCC post-platinum	Nivolumab	Standard chemo	OS 7.5 vs. 5.1 months (HR 0.70)	Second-line standard
QUARTERBACK 2b	Phase II	LA HPV+ (n = 50)	Induction chemo → reduced RT	Historical control	2-year PFS 85%	Sequential de-escalation

## Data Availability

No new data were created or analyzed in this study.
